# An Experimental Study on the Effectiveness of Disclosing Stressful Life Events and Support Messages: When Cognitive Reappraisal Support Decreases Emotional Distress, and Emotional Support Is Like Saying Nothing at All

**DOI:** 10.1371/journal.pone.0114169

**Published:** 2014-12-22

**Authors:** Anika Batenburg, Enny Das

**Affiliations:** 1 Department of Communication Science, Faculty of Social Sciences, VU University Amsterdam, Amsterdam, the Netherlands; 2 Centre for Language Studies, Radboud University Nijmegen, Nijmegen, the Netherlands; George Mason University, United States of America

## Abstract

How can we best support others in difficult times? Studies testing the effects of supportive communication revealed mixed findings. The current study focuses on the effects of supportive communication following different disclosure styles, and includes outcome measures to assess emotional well-being. Hypotheses were tested in a 2 (disclosure style: cognitive reappraisal disclosure *vs.* emotional disclosure) ×3 (support message: cognitive reappraisal response *vs.* socio-affective response *vs.* no response) between subjects factorial design. Receiving a cognitive reappraisal response, rather than a socio-affective response or no response, decreased emotional distress in the emotional disclosure group. Support messages showed no effects in the cognitive reappraisal disclosure group. Although socio-affective responses were positively evaluated, cognitive reappraisal responses may be more effective during emotional upheaval because they provide a positive way out of negative emotions.

## Introduction

A little comfort can go a long way during moments of distress. Research has shown that social support may improve coping with stressful events, positively affect relationships, and decrease levels of emotional distress (for an overview of literature, see [1]). However, the question remains: what do we need to say to let others benefit most from our support? Is it most important to acknowledge and understand ones' feelings or should we help the person to change perspective by portraying it as a learning experience and focusing on the future?

The current study has an interdisciplinary character by combining knowledge from two fields of research; communication research on support messages and social psychology literature on processing and disclosing trauma. We propose that effects of a support message might depend on the disclosure style of the individual in need. Previous research showed that the psychological impact of an event depends not only on the type of support individuals receive, but also on one's personal appraisal of the experience [2], [3]. Psychological research suggests that after a traumatic or stressful experience individuals go through different phases of appraisal and emotional arousal, and these phases influence one's needs for support [4]. In line with these findings, we put forward that support messages should match individual's disclosure style.

Furthermore, we aim to extend previous research on support communication by assessing effects of social support messages not only by indications of self-reported helpfulness, but also with regard to emotions and emotion-related symptoms. Most previous studies on support messages assessed the effectiveness of support messages by self-reported evaluations of helpfulness or perceived affective change. However, perceptions of helpfulness do not necessarily correlate with actual emotional distress relief [5], [6], [7]. In order to move research in this domain beyond indications of what individuals think a conversational partner should say, we aim to compare these with actual psychological emotional distress measures in the present study.

The next section starts by providing an overview of empirical research on supportive communication. We then forward several propositions regarding the interaction between disclosure style and supportive communication, followed by a discussion on the reliance on introspective outcome measures. We describe an experimental study to test the effects of the fit between disclosure style and support message on both perceptions of helpfulness (i.e., evaluations of appropriateness, pleasantness, and supportiveness) and measures of emotional distress (i.e., emotions and emotion-related symptoms).

### Supportive communication

What makes supportive communication effective? Research examining this question has increased our understanding extensively by assessing the type of support provided and its perceived helpfulness in conversations about a stressful event [8]. However, some findings across studies appear mixed, e.g., [8]–[13]. The research field mainly consists of two types of approaches. Departing from a *naturalistic framework* [14], [15], descriptive typologies of support behaviors were developed based on retrospective self-reports. In these retrospective self-reports, individuals are asked to memorize the responses they received from others following a stressful life event and evaluate the helpfulness of each response, e.g., [16]–[18]. This approach has yielded insight into helpful and unhelpful behaviors. For example a study on cancer patients classified ‘emotional support behaviors’, ‘being physically present’, and ‘showing empathy and concern’ as helpful behaviors, and ‘critical responses’ or ‘minimization’ as unhelpful behaviors [17]. The difficulty is, however, that different contexts have generally yielded different typologies, and therefore findings are not easily generalized across different situations.

Research based on the deductive *message perception paradigm* [14], [15] tested perceptions of helpfulness of pre-defined support messages across contexts. In this research paradigm, the researcher presents an imaginary scenario or dialogue (see [19] for an exception that deals with actual experienced situations), followed by different, often emotional, support messages. Participants are asked to indicate the helpfulness, effectiveness, appropriateness or sensitivity of each support message, e.g., ([20] (Study 2) [21], [22]. Across studies, this paradigm has also yielded different results; for instance, giving advice is in some situations perceived as helpful, whereas in others it is not.

To overcome these mixed findings, some researchers proposed ‘matching models’ according to which supportive interactions should match coping demands created by a certain stressor. For example, Cutrona and colleagues distilled five types of support: emotional support; network support; esteem support; tangible support; and informational support [23]–[25] (for a slightly different model see [26]), and four dimensions of life stressors; desirability (i.e., intensity of negative emotions the event provokes), controllability (i.e. preventability of the consequences of the event), duration of the consequences, and its life domain (i.e., loss or treat of assets, relationships, achievements, social roles [23]). They propose that support type should match the demands produced by the stressful event. A number of studies indeed found the proposed effects, e.g., [11], [12], [27]. However, others did not, e.g., [9], [28], [13].

### Disclosure style

One reason for observed inconsistencies in findings across studies may be that most studies focused on characteristics of the event (as categorized by the researcher) and the type of support received, but did not take into account individual differences in appraisal and disclosure style. These might however be of interest, considering that individuals who experience a negative event use different emotion regulation strategies [29], and have their own interpretation of its emotional load, controllability, and consequences [2]. Although to our knowledge the matching between support type and disclosure style has not received any empirical attention, Jacobsen already underscored the necessity of a match between support messages and phase of disclosure in 1986 [30]. He suggests that support should match ‘stressor sequences’ [31]. Specifically, a crisis situation (i.e., when something occurs or changes abruptly that elicits emotional arousal) especially demands emotional support, whereas in times of transition (i.e., a period of personal and relational change between the individual and the stressor) cognitive support is more appropriate, and in a deficit state (i.e., a situation in which someone's life is defined by chronically excessive demands) someone is in need of material support and direct action to restore the balance between needs and tangible resources. Related to this point, Rimé has proposed that coping with stressful events includes different regulation needs; socio-affective needs (i.e., emotional support, comforting) during the emotional episode, cognitive needs (i.e., reorganization of motives, re-creation of meaning) to overcome perseveration, and action needs in the form of creating new experiences [4]. Hence, since processing a stressful life experience follows a sequence of different coping phases, like Jacobsen (1986) suggested, we propose that support messages are required to match the current appraisal of the person in need.

Although until now this proposition has not been tested explicitly in the context of supportive communication, more information regarding the effects of disclosing stressful life events can be found in the expressive-writing literature. Expressive writing is a form of expressive therapy aimed to help individuals to overcome emotional trauma. In expressive writing experiments, participants express their deepest thoughts and feelings about a stressful event that has affected them and their life (for the explicit assignment, see [32]). Research has shown that such disclosure about emotional life events positively affects psychological and physical health over time, e.g., [32]–[37]. In line with the idea of Jacobsen and Rimé that processing a stressful event follows a sequence of different phases and needs, Lepore. Greenberg, Bruno, and Smyth suggested that expressive writing enables three important underlying mechanisms to cope with trauma; directing attention to the stressor and related emotions, habituation to the emotions, and cognitive restructuration [38]. Especially cognitive restructuring the experience appears of value in this psychological process since the influence of stress on health outcomes is mediated by appraisal [2]. Hence, expressive writing initially promotes habituation to emotions and coping with demands related to the stressor, and in turn there is mental capacity to positively reinterpret the stressor and its relation to the self. Therefore emotional disclosure seems to facilitate cognitive reappraisal [39].

In an experimental test of this idea, Lu and Stanton used different disclosure assignments, focused on emotional disclosure, cognitive reappraisal, or a combination of both [39]. With the emotional disclosure instructions, participants had to focus on their deepest emotions about a current most stressful experience that had affected them and their lives. The cognitive reappraisal assignment was mainly focused on perceptions of the stressful event, consequences of the event, challenges and opportunity arising from the event, and cognitive reappraisal of coping strategies. Results revealed that cognitive reappraisal writing reduced physical symptoms, emotional disclosure buffered a decrease in positive affect over time, and the combination of emotional disclosure and cognitive reappraisal was most effective on both physical symptoms and positive affect.

However, to date no study has tested what type of social support is the most valuable when individuals are emotionally aroused by thinking about the experience (i.e., crisis situation) or when they are cognitively restructuring the event (i.e., in times of transition). We propose that support is most effective when it matches disclosure style of the recipient. The first goal of the present study was thus to empirically test the proposition that social support messages should fit the recipient's disclosure style. Based on the above reasoning, we propose that individuals with an emotional disclosure style benefit especially from a socio-affective support message, and that individuals with a cognitive reappraisal disclosure style benefit most from a cognitive reappraisal support message **(main hypotheses)**.

### Evaluations of helpfulness

The second goal of this study is to extend previous studies by testing the effects of support messages by assessing participants' emotions, in addition to self-reported perceptions of helpfulness. Thus far, most studies assessed the effectiveness of social support messages using self-report ratings of helpfulness (or sometimes ‘sensitiveness’, ‘supportiveness’, ‘appropriateness’, ‘effectiveness’; e.g., [8], [40]) or perceived affective improvement, e.g., [19], [41], [42]. These studies have increased our knowledge on support messages but introspective procedures have their limits, simply because not all mental processes are accessible to people. For instance, when individuals are asked to report why they made a certain choice or how they arrived at a certain judgment, the resulting reports are often confabulated [5], [6]. People may underestimate the helpfulness of unpleasant strategies in particular. For instance, a study on public speaking showed that talking about feelings was related to less fear of speaking, but was not related to self-reported supportiveness [7]. Hence, although individuals may perceive some types of support as less- or unhelpful, there are conditions under which this support may still be good for them, i.e., have a positive impact on their emotional well-being. This may hold true especially for socially undesirable support strategies. For example, socio-affective responses in which a conversational partner affirms an individual's emotions may positively affect perceptions of relatedness to the response provider but may not necessarily be most beneficial in terms of emotion and health outcomes.

The current study is a first attempt to increase insight into the effects of social support by including evaluations of the support message as well as relatedness to the support provider, and measures of emotional well-being, i.e., emotions and emotion-related symptoms [43]. Since there is a lack of knowledge on the relationship between support message evaluations (i.e., appropriateness, pleasantness, supportiveness), relatedness to the support message provider, and emotional well-being in the context of support messages, we introduce a guiding research question **(RQ)**: What is the relationship between perceptions of helpfulness, relatedness and emotional distress, and is this relationship moderated by the match of disclosure style and support message?

### Overview

Previous studies have investigated supportive communication, but the match with individual's disclosure style has not been examined and findings beyond self-reported perceptions of helpfulness are lacking. We propose an experiment to test the combined effects of *disclosure style* (emotional disclosure *vs.* cognitive reappraisal) and *support messages* (cognitive reappraisal (CR) response *vs.* socio-affective (SA) response *vs.* no response) on *support message evaluations* (i.e., appropriateness, pleasantness, and supportiveness); the extent to which one *feels related to the response provider*; *emotions*; and *emotion-related symptoms*.

## Method

### Design and Participants

Hypotheses were tested in a 2 (Disclosure style: cognitive reappraisal *vs.* emotional disclosure) ×3 (Support message: cognitive reappraisal (CR) response *vs.* socio-affective (SA) response *vs.* no response) between subjects factorial design. There were 122 individuals who participated in this study. Most of them were undergraduate students and received credits for participation. Seven respondents were excluded from data analysis because they misunderstood the disclosure assignment. Our sample consisted of 115 respondents (87 females and 28 males), with a mean age of 22 years (*SD* = 8.42). The distribution of male and female participants was almost equal per experimental condition (emotional disclosure style, 14 males and 40 females; cognitive reappraisal disclosure style, 14 males and 47 females; no response, 9 males and 29 females; SA response, 10 males and 32 females; CR response, 9 males and 26 females).

### Procedure and Independent Variables

All respondents were invited to participate in a study about written disclosure. Half the respondents received disclosure instructions focused on emotional expression and the other half received instructions facilitating cognitive reappraisal (for the exact writing instructions, see [39]). The emotional disclosure group was instructed to write 15 minutes about their deepest emotions about a current most stressful event that affected them and their lives. They were asked to let go and explore their feelings and thoughts about it. Participants assigned to the cognitive reappraisal condition were instructed to write 15 minutes about positive and negative consequences of a current most stressful event, their perceptions of the stressful event, challenges and opportunity arising from the event, cognitive reappraisal of their coping strategies and their positive thoughts about the stressor. After the disclosure assignment participants were first told that another respondent would read and react on their story (only in the conditions where participants received a SA or CR response) and then answered filler questions and filled out demographics, to make it plausible that another participant had enough time to read and respond on their story in the meantime.

Subsequently, respondents randomly received a response to their story on their computer screens (except for the control group, who received no response), purportedly from another anonymous participant. This response was manipulated as a socio-affective response or a cognitive reappraisal response. Responses were matched according to length and valence in ‘person centeredness’, i.e., the extent to which the feelings and perspective of a distressed other are explicitly acknowledged, elaborated, and granted legitimacy [8]. The difference in response type (socio-affective response *vs.* cognitive reappraisal response) was based on the regulation needs of Rimé, whereby the socio-affective response is especially focused on social integration by comforting, understanding and legitimating feelings [4]. Participants in the socio-affective response condition read the response: ‘*Dear writer, thanks for telling me your story. I think it was an impressive story. It must have been intense to experience something like that. I experienced something quite similar, and I recognize a lot in your story. I understand how it must have felt and the impact it must have had on your life. Take care.’* The cognitive reappraisal response, in contrast, focused on the recreation of meaning, i.e., learning from- and coping with the experience in order to change motives or goals. Respondents in the cognitive reappraisal response condition read: *‘Dear writer, thanks for telling me your story. I admire the way you dealt with this situation. Learning from these experiences is very important. Whenever you will experience something similar, you know better how to deal with it. I wish you good luck in the future.’* After they received this support message, we measured participants' emotions and emotion-related symptoms. Subsequently, except for the control group, participants evaluated the support message they received (i.e., appropriateness, pleasantness, supportiveness) and if they felt related to the anonymous person that provided the support message.

### Manipulation Checks

#### Disclosure assignment

To confirm that the two different writing assignments elicited a different disclosure style, the stories participants wrote during the experiment were analyzed with the Dutch LIWC computerized text analysis program [44], [45]. The software is designed to analyze written text on a word-by-word basis. The program calculates the percentage of words in the text that matches different language dimensions, such as emotional, cognitive, structural, and process components. The proportion of words indicating each dimension was counted for each participant. One would expect that the cognitive reappraisal disclosure assignment should elicit the use of more *cognitive mechanism* words (words indicating causation, e.g. *because, depend;* insight, e.g. *know, explain;* discrepancy, e.g. *should, would;* inhibition, e.g. *block, conflict;* tentativeness, e.g. *perhaps, might;* and certainty, e.g., *always, never*) than the emotional disclosure assignment, and that the emotional disclosure assignment should bring forward the use of more words indicating *negative emotions* (e.g. *sad, hate, hurt, guilty*) (word categories LIWC; [44], [45]) than the cognitive reappraisal assignment. Previous studies support the reliability and validity of LIWC-based analyses, e.g., [46], [47].

#### Support message

To verify if the social support responses differed in socio-affective level, three items measured *perceived socio-affective characteristics* (validating, soothing, comforting; Cronbach's α = .86). For example, ‘The response from the other person was comforting?’.

### Dependent Measures

#### Emotions

Emotions were measured with the Symptom/emotion checklist: a state measure [43], including 5 items (e.g., sad) on a 5-point scale (Cronbach's α = .83). Positive emotion items were recoded. Higher scores imply more negative emotions.

#### Emotion-related symptoms

A 12-item symptom measure (Symptom/emotion checklist: a state measure [43]) was used to assess *emotion-related symptoms* respondents felt after disclosing their story and receiving the support message. Participants rated on a 5-point scale if they felt the symptoms or not (‘Now, at this moment, I have a headache’; Cronbach's α = .81). Ratings were summed and averaged across items. Higher scores indicate more emotion-related symptoms.

#### Support message evaluation

Three items were included to assess *response evaluation* (appropriateness, pleasantness, supportiveness; Cronbach's α = .87). In previous studies single-item outcome variables have frequently been used to measure message quality, for example by appropriateness, effectiveness, or supportiveness [21], [22]. Item example; ‘did you perceive the reaction of the other person to your story as supportive?’. All items were answered on a 5-point scale from ‘Not at all’ to ‘Very much’.

#### Perceived relatedness

Participants filled out a 4-item measure on a 4-point scale to assess perceived relatedness to the person who wrote the response (e.g. ‘I feel that I associate with the person who read and responded to my story, in a very friendly way’). These questions were based on the relatedness subscale in the Autonomy, Competence, and Relatedness in Exercise scale [48]. The scale was internally consistent (Cronbach's α = .85). See S1 Appendix for the items of all dependent variables.

### Covariates

Because it is plausible that a very recent event has more impact on well-being than something that happened years ago, participants were asked when the event occurred. Participants could respond by choosing one of six categories, ranging from ‘this year’ to ‘more than 8 years ago’. For 35,7% of the participants the event took place last year, for 15,7% about a year ago, for 14,8% about two years ago, for 13,9% about 3 or 4 years ago, for 12,2% about 5 till 8 years ago, and for 7,8% more than 8 years ago.

To examine a potential influence of the topic participants wrote about, all stories were coded by its' subject. The first author coded the stories based on the Life Events Inventory [49], in which life events are ranked for the severity of the stress they elicit. The second author coded 50% of the stories to test for inter-coder reliability, which was high (Kalpha  = .94). Since most of our participants were undergraduate students, ranking was based on results of LEI scales tested among student samples [50], [51]. See S2 Appendix for the codebook.

### Ethics Statement

All procedures were approved by the Department of Communication Science of the VU University Amsterdam, because 1) no adverse events were expected based on the current expressive writing literature, 2) experimental conditions do not deviate from participants' real life situations, 3) participants voluntarily chose the topic they wrote about and where in control of the details they disclosed. The study adhered to all the APA ethical guidelines [52], and complies with EU legislation [53] and the Dutch legislation [54] on data protection. Participants (mostly undergraduate students) voluntary registered online to participate in the study to earn credits. On this university website, students can freely pick a study that appeals to them out of a number of studies provided. The online introduction page of the experiment included the length and purpose of the study (i.e., writing about a personal distressful life event, and that during the study there was a possibility that another study participant would read the story written) contact information of the investigator (in case participants would have any questions), and ensured anonymity. On the last page of the study, participants were debriefed; we explained that we were examining the effects of support messages, and that the response of the other study participant was automated, hence not real, and that no other participant read the story written. We again provided them with contact information on the last page, in case participants would have any additional questions.

## Results

### Manipulation Checks

#### Disclosure assignment

A unifactor (disclosure condition: emotional disclosure *vs.* cognitive reappraisal disclosure) ANOVA revealed the expected difference in the use of *negative emotion* words and *cognitive mechanism* words between the two disclosure assignments. Participants in the emotional disclosure condition used more negative emotion words (*M* = 2.72, *SD* = 0.89) than participants in the cognitive reappraisal disclosure condition (*M* = 2.16, *SD* = 0.89), *F*(1,113) = 11.184, *p*  = .001, *η^2^_ρ_*  = .090. Results also showed that participants used more cognitive mechanism words in the cognitive reappraisal disclosure condition (*M* = 6.89, *SD* = 1.56), than participants in the emotional disclosure condition (*M* = 6.22, *SD* = 1.57), *F*(1,113) = 5.210, *p* = .024, *η^2^_ρ_* = .044.

#### Support message

A unifactor (support message condition: socio-affective response *vs*. cognitive reappraisal response) ANOVA on *perceived socio-affective characteristics* showed that the socio-affective response (*M* = 2.94, *SD* = 1.17) was perceived as significantly more socio-affective (i.e., soothing, comforting, validating) than the cognitive reappraisal response (*M* = 2.37, *SD* = 1.06), *F*(1,73) = 4.840, *p*  = .031, *η^2^_ρ_* = .062.

### Effect testing

Correlation analyses between all dependent variables showed that there was a significant relation between emotions and emotion-related symptoms, and between support message evaluation and perceived relatedness (see Table 1).

**Table 1:** Correlations between Dependent Variables.

DV	1	2	3	4	5	6
1. Negative emotion words (LIWC)	-					
2. Cognitive mechanism words (LIWC)	.013	-				
3. Emotions	.139	−.041	-			
4. Emotion-related symptoms	.091	−.093	.629**	-		
5. Message evaluation	.102	.117	.094	.108	-	
6. Relatedness	.132	.047	.126	.142	.606**	-

*Note*. **p<.001.

#### Support message evaluation

A 2 (disclosure condition: cognitive reappraisal *vs.* emotional disclosure) by 2 (support message condition: cognitive reappraisal *vs.* socio-affective) ANOVA on *support message evaluation* (i.e., appropriateness, pleasantness, supportiveness) revealed no effect of support message (socio-affective response *vs*. cognitive reappraisal response; *F*<1); disclosure style (cognitive reappraisal disclosure *vs.* emotional disclosure; *F*<1) or an interaction effect of the disclosure condition and the support message condition (*F*<1; see Table 2). Participants thus perceived the two different support messages as equally appropriate, pleasant and supportive (socio-affective response; *M* = 3.21, *SD* = 1.17; cognitive reappraisal response; *M* = 3.17, *SD* = 1.11).

**Table 2:** Disclosure Condition x Support Message Condition Factorial Analysis of Variance for Message Evaluations.

Source	*Df*	*F*	η2	*p*
(A) Disclosure Condition	1	.05	.001	.822
(B) Support Message Condition	1	.00	.000	.948
A×B (interaction)	1	.11	.002	.737
Error (within groups)	71			

#### Perceived relatedness

A 2×2 ANOVA showed a marginally significant main effect of the support message condition on relatedness to the person who provided this message, *F*(1,71) = 3.30, *p* = .073, *η^2^_ρ_* = .044. Respondents felt slightly more related to the person who provided the socio-affective response (*M* = 2.73, *SD* = 1.04) than to the person who provided the cognitive reappraisal response (*M* = 2.28, *SD* = 0.79). No significant main effect of disclosure condition (*F*<1) and no interaction was found (*F*(1,71) = 1.60, *p*  = .210, *η^2^_ρ_* = .022; see Table 3).

**Table 3:** Disclosure Condition x Support Message Condition Factorial Analysis of Variance for Relatedness.

Source	*Df*	*F*	η2	*p*
(A) Disclosure Condition	1	.00	.000	.977
(B) Support Message Condition	1	3.33	.044	.073
A×B (interaction)	1	1.60	.022	.210
Error (within groups)	71			

#### Emotions

A 2×3 ANOVA revealed a main effect of the disclosure condition on emotions, *F*(1,109) = 5.71, *p* = .019, *η^2^_ρ_* = .050. Participants assigned to the cognitive reappraisal disclosure condition experienced less negative emotions (*M* = 1.77, *SD* = 0.55) than respondents in the emotional disclosure condition (*M* = 2.14, *SD* = 0.88). Furthermore, a significant interaction effect of disclosure condition and support message condition on emotions was observed, *F*(2,109)  = 3.70, *p* = .028, *η^2^_ρ_* = .064 (see Table 4).

**Table 4:** Disclosure Condition x Support Message Condition Factorial Analysis of Variance for Emotions.

Source	*Df*	*F*	η2	*p*
(A) Disclosure Condition	1	5.71	.050	.019
(B) Support Message Condition	2	1.45	.026	.239
A×B (interaction)	2	3.70	.064	.028
Error (within groups)	109			

**Figure 1:**
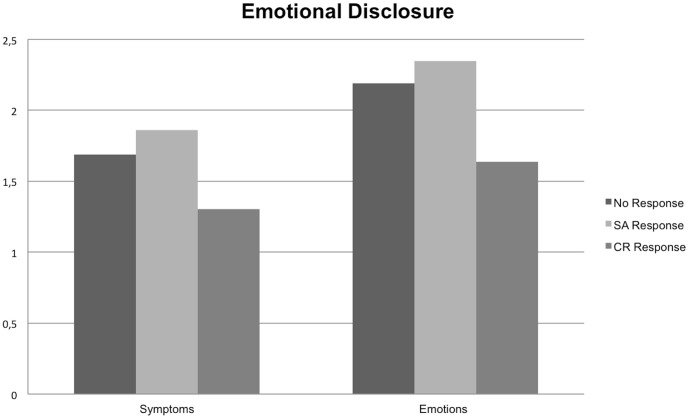
Emotional Disclosure.

**Figure 2:**
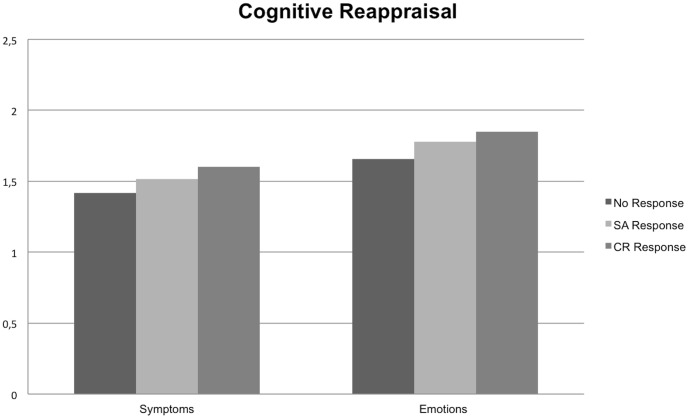
Cognitive Reappraisal.

Post-hoc comparisons indicated that significant mean differences emerged for respondents in the emotional disclosure condition. Respondents reported less negative emotions after a cognitive reappraisal response (*M* = 1.64, *SD* = 0.62) compared with a socio-affective response (*M* = 2.35, *SD* = 0.96; *p* = .006), or no response (*M* = 2.19, *SD* = 0.83; *p* = .037). The difference between the socio-affective and control condition was not significant (Fig. 1). No significant simple effects in the cognitive reappraisal disclosure condition were found (Fig. 2).

#### Emotion-related symptoms

A 2×3 ANOVA revealed only an interaction effect of disclosure condition and support message condition on emotion-related symptoms, *F*(2,109) = 3.30, *p* = .041, *η^2^_ρ_* = .057 (See Table 5).

**Table 5:** Disclosure Condition x Support Message Condition Factorial Analysis of Variance for Emotion-related Symptoms.

Source	*Df*	*F*	η2	*p*
(A) Disclosure Condition	1	1.02	.009	.316
(B) Support Message Condition	2	1.50	.027	.227
A×B (interaction)	2	3.30	.057	.041
Error (within groups)	109			

Post-hoc comparisons indicated that significant mean differences emerged for respondents in the emotional disclosure condition; respondents reported less symptoms after the cognitive reappraisal response (*M* = 1.30, *SD* = 0.33) compared with the socio-affective response (*M* = 1.86, *SD* = 0.74; *p* = .008) or no response condition (*M* = 1.69, *SD* = 0.72; *p* = .071), although the latter effect was only marginally significant. The difference between the socio-affective response and no response condition was not significant (Fig. 1). No significant simple effects were observed in the cognitive reappraisal writing condition (Fig. 2).

### Additional analyses

To reveal if the topic participants wrote about or the time since the event happened had an influence on the dependent variables (i.e., emotions, emotion-related symptoms, support message evaluation and perceived relatedness) we ran a correlation matrix. Only the topic of the story was related to emotions, no other correlations were found. The more serious the topic (i.e., the lower the score on this variable) the more negative emotions participants experienced (*r* = −.208, *p* = .025). We added ‘story subject’ to our model to see if this would change our findings. The 2 (disclosure condition: cognitive reappraisal *vs.* emotional disclosure) by 3 (support message condition: cognitive reappraisal *vs.* socio-affective *vs.* no response) ANOVA still revealed a similar main effect of the assignments on emotions, *F*(1,108) = 4.65, *p*  = .033, *η^2^_ρ_* = .041. The previous found interaction effect of disclosure condition and support message condition on emotions became marginally significant, *F*(2,108)  = 2.91, *p*  = .059, *η^2^_ρ_* = .051. Post-hoc comparisons showed exactly the same mean differences as before; respondents reported fewer negative emotions after a cognitive reappraisal response (*M* = 1.64, *SD* = 0.62) compared with a socio-affective response (*M* = 2.35, *SD* = 0.96; *p* = .015), or no response (*M* = 2.19, *SD* = 0.83; *p* = .050). No main effect of ‘story subject’ on emotions was found.

## Discussion

The present study tested the effects of disclosing a negative life experience and receiving a supportive response on perceived helpfulness, relatedness to the support message provider, emotions and emotion-related symptoms of the recipient. Supportive responses moderated the effects of disclosure style on emotions and emotion-related symptoms. Cognitive reappraisal responses, which focused on reinterpreting the negative life experience, decreased negative emotions and symptom reporting particularly for individuals who had just expressed their deepest emotions, i.e., for participants in the emotional disclosure condition. Supportive responses had no effect on participants who disclosed a negative life event by cognitively reappraising the experience.

These findings suggest that cognitively reappraising a stressful situation may have beneficial effects on well-being in two different ways. First, the fact that individuals who cognitively reappraised a stressful situation had similar – lower – levels of negative emotions and emotion-related symptoms regardless of type of support message they received suggest that cognitively reappraising a negative life experience makes individuals less vulnerable to responses from others. Cognitively re-evaluating a negative experience might not only make individuals feel better about the situation, it also buffers ones susceptibility to responses. Cognitive reappraisal may thus promote resilience and a decreased dependency on others.

Second, cognitive reappraisal responses from a conversational partner may help individuals to interpret an emotional experience from a different viewpoint, especially when they are emotional; it might provide a positive way out of negative emotions. Solely disclosing emotions attached to a stressful situation could evoke a vicious cycle of negative emotions, which may drain individual resources to look at a situation from a different viewpoint. In such conditions, supportive responses may be helpful to break this vicious cycle and help individuals see a different picture. These findings are in line with Rimé and, Lu and Stanton, who proposed that satisfaction of socio-affective needs is not sufficient; individuals should fulfill their cognitive needs as well to overcome mental rumination and intrusive thoughts [4], [39].

Furthermore, studies showed that individuals who reappraise stressful situations innately (i.e., “*constructing a more positive meaning out of the many possible meanings that may be attached to that situation*” p.352, [29] generally show more positive emotions, fewer negative emotions, and a better well-being [3], [55] than individuals with a lower score on this regulation strategy. Thus support messages that stimulate to cognitively reappraise the situation might help individuals to change perspective, especially when individuals do not naturally use reappraisal as emotion regulation strategy. In future studies it might be interesting to assess if individual differences in ingrained use of certain emotion regulation strategies (e.g., reappraisal, suppression) affect the current effects.

Contrary to expectations, our findings suggest conditions under which responses that do not match a certain style of disclosure are actually better than matched responses, and that validating one's negative feelings does not break the vicious cycle of negative emotions. Future studies should further examine effects of different support messages on well-being, for example by comparing short versus long-term effects of different disclosure styles and support types on well-being. There is some empirical evidence that expressing one's emotions elicits more emotional distress and a higher heart rate during disclosure, but promotes psychological well-being in the longer run [56], [57]. It should be worthwhile examining whether diminishing negative emotions by providing cognitive reappraisal support messages also promotes long-term well-being.

The present study also extends previous research on supportive communication by comparing effects on emotional distress to the evaluation of the support message. This study seems to indicate that individuals are not always capable of assessing certain effects on their own well-being. Participants felt slightly more related to the person who provided a socio-affective response, and perceived this response as more soothing, comforting, and validating than a cognitive reappraisal response. However, these positive evaluations did not translate into lower levels of emotional distress. On the contrary, participants who just expressed their deepest emotions did not benefit from a socio-affective response; levels of emotional well-being were similar to the control condition (i.e., no response), and lower than the cognitive reappraisal response condition. Finally, although the experimental conditions showed no effects on perceived supportiveness of the support message, effects were observed on measures of emotional well-being. Additionally, message evaluations were unrelated to emotions, and to emotion-related symptoms. Together, these findings indicate the need for additional outcome measures next to self-perceived helpfulness in future studies.

### Limitations and Future Research

A limitation of this research is that only two different response messages were used to cover different response types. For example, Jackson and Jacobs recommend using more than one message to cover a support category in order to verify whether the different support messages differ in the proposed theoretical categories, or whether there was something particular about the messages that led to the observed effects [58]. To keep the experiment as naturalistic as possible we chose to provide participants with only one supportive response purportedly from another study participant. Nonetheless, one message to cover a response type is limited, and in future research experiments should be extended with more responses that cover one response type.

A second limitation is the lack of a control group for the writing assignment, i.e., study participants who write about a neutral event. Since we were especially interested in the effects of different support messages when individuals disclose stressful events, we only included a control group for the support message condition and did not include a control group for the writing assignment. In future research it might be interesting to compare the effects of the different writing assignments in order to gain a better understanding of baseline values for the measures used in the present research.

Furthermore, we cannot exclude the possibility of selection bias. For ethical reasons we had to inform potential participants upfront that they would disclose a personal stressful life event. There is a possibility that the current study participants differ from individuals not willing to participate. For example, the current participants might have a higher need for disclosure (i.e., to talk about thoughts and feelings) than individuals who decided not to participate, and that, in turn, might have had an influence on the effects of the support messages.

Another restriction is that an extensive part of the participants were females. Although there was no effect of gender on the dependent variables and every experimental condition contained an almost equal distribution of males and females, it could be that gender has an effect on moderators of the psychological process, such as personality traits or coping strategies. For example, a meta-analysis focused on gender differences in coping showed that females cope by engaging in social relationships and they try to create change (in cognitive and actual terms) more frequently than men do. On the other hand, males rely more often on stress reduction activities or they tend to distract themselves (i.e., diversions) [59]. Gender differences may be important for the process of recovering from a stressful event, and should be further investigated in relation to social support messages.

Additionally, in the current study the response provider was an unknown anonymous person. Future research should reveal if responses from significant others (e.g., family, friends) elicit different outcomes. Finally, future studies should examine long-term effects on well-being. By repeating this experiment and conducting additional measurements for emotional distress or well-being a few weeks later, it may be possible to see how disclosure in combination with different support messages affects well-being over time.

### Conclusions

The current study findings suggest that responding by cognitively reappraising a stressful situation may produce positive effects on emotions and emotion-related symptoms. Although telling someone that ‘you understand how they feel’ is perceived as helpful and might increase a relational bond, it may not be the best strategy to get someone back on track following a stressful situation: in the current study its effects are similar to saying nothing at all.

## Supporting Information

S1 Appendix **Survey questions outcome measures.** Symptom/emotion checklist: a state measure (Pennebaker, 1982).
(DOCX)

S2 Appendix **Codebook Story Subjects.**
(DOCX)

## References

[1] Cunningham MR, Barbee AP (2000) Social support. In: Hendrick C, Hendrick SSeditors.Close relationships: A sourcebook. Thousand Oaks, CA: Sage. pp.272–285.

[2] Lazarus RS, Folkman S (1984) Stress, appraisal, and coping. New York: Springer.

[3] Gross JJ, John OP (2003) Individual differences in two emotion regulation processes: Implications for affect, relationships, and well-being. J Pers Soc Psychol 85:348–362 doi:10.1037/0022-3514.85.2.348. 12916575

[4] Rimé B (2007) Interpersonal emotion regulation. In: Gross JJeditor.Handbook of emotion regulation. New York: The Guilford Press. p. 466–485.

[5] Nisbett RE, Wilson TD (1977) Telling more than we can know: Verbal reports on mental processes. Psychol Rev 8:231–259.

[6] Johansson P, Hall L, Sikström S, Tärning B, Lind A (2006) How something can be said about telling more than we can know: On choice blindness and introspection. Conscious Cogn 15(4):673–692 doi:10.1016/j.concog.2006.09.004. 17049881

[7] Winstead BA, Derlega VJ, Lewis RJ, Sanchez-Hucles J, Clarke E (1992) Friendship, Social Interaction, and Coping With Stress. Communic Res 19(2):193–211 doi:10.1177/009365092019002004.

[8] Burleson BR, Goldsmith DJ (1998) How the comforting process works: Alleviating emotional distress through conversationally induced reappraisals. In: Andersen PA, LK Guerreroeditors.Handbook of communication and emotion: Research, theory, applications, and contexts. San Diego, CA, US: Academic Press. pp.245–280.

[9] Baker JM (1997) Social support and academic persistence: A test of the optimal-matching model. Unpublished dissertation, Arizona State University, Tempe, AZ.

[10] Burleson BR (2009) Understanding the outcomes of supportive communication: A dual-process approach. J Soc Pers Relat 26(1):21–38 doi:10.1177/0265407509105519.

[11] Kaniasty K, Norris FH (1992) Social support and victims of crime: Matching event, support, and outcome. Am J Community Psychol 20:211–241 doi:10.1007/BF00940837. 1605134

[12] Pierce LI, Frone MR, Russel M, Cooper ML (1996) Financial stress, social support, and alcohol involvement: A longitudinal test of the buffering hypothesis in a general population survey. Health Psychol 15:38–47 doi:10.1037/0278-6133.15.1.38. 8788539

[13] Wade TD, Kendler KS (2000) Absence of interactions between social support and stressful events in the prediction of major depressive symptomatology in women. Psychol Med 30:965–974 doi:10.1017/S0033291799002251. 11037104

[14] Burleson BR, MacGeorge EL (2002) Supportive communication. In: Knapp ML, Daly JAeditors.Handbook of interpersonal communication (3rd ed.).Thousand Oaks, CA: Sage. pp.374–422.

[15] Jones SM (2004) Putting the person into person-centered and immediate emotional support: Emotional change and perceived helper competence as outcomes of comforting in helping situations. Communic Res 31(3):338–360 doi:10.1177/0093650204263436.

[16] Costanza RS, Derlega VJ, Winstead BA (1988) Positive and negative forms of social support: Effects of conversational topics on coping with stress among same-sex friends. J Exp Soc Psychol 24:182–193 doi:10.1016/0022-1031(88)90020-0.

[17] Dakof GA, Taylor SA (1990) Victim's perceptions of social support: what is helpful from whom? J Pers Soc Psychol 58:80–89 doi:10.1037/0022-3514.58.1.80. 2308075

[18] Lehman DR, Hemphill KJ (1990) Recipients' perceptions of support attempts and attributions for support attempts that fail. J Soc Pers Relat 7:563–574 doi:10.1177/026540759007401.

[19] Jones SM, Guerrero LK (2001) The effects of nonverbal immediacy and verbal person centeredness in the emotional support process. Hum Commun Res 27:567–596 doi:10.1111/j.1468-2958.2001.tb00793.x.

[20] Burleson BR, Samter W (1985) Consistencies in theoretical and naïve evaluations of comforting messages. Commun Monogr 52:125–153 doi:10.1080/03637758509376099.

[21] Goldsmith D, MacGeorge E (2000) The impact of politeness and relationship on perceived quality of advice about a problem. Hum Commun Res 26:234–263 doi:10.1111/j.1468-2958.2000.tb00757.x.

[22] Jones SM, Burleson BR (1997) The impact of situational variables on helpers' perceptions of comforting strategies. Communic Res 24:530–555 doi:10.1177/009365097024005004.

[23] Cutrona CE, Russel DW (1990) Types of social support and specific stress: Towards a theory of optimal matching. In: Sarason BR, Sarason IG, Pierce GReditors.Social support: An interactional view. New York: Wiley. pp.319–366.

[24] Cutrona CE, Suhr JA (1992) Controllability of stressful events and satisfaction with spouse support behaviors. Communic Res 19(2):154–174 doi:10.1177/009365092019002002.

[25] Cutrona CE, Suhr JA (1994) Social support communication in the context of marriage: An analysis of couples' supportive interactions. In: Burleson BR, Albrecht TL, Sarason IGeditors.Communication of social support: Messages, interactions, relationships, and community.Thousand Oaks, CA: Sage Publications. pp.113–135.

[26] Cohen S, McKay G (1984) Social support, stress and the buffering hypothesis: A theoretical analysis. In: Baum A, Taylor SE, Singer JEeditors.Handbook of psychology and health: Vol. IV. Social psychological aspects of health.Hillsdale, NJ : Erlbaum.pp.253–267.

[27] Swanson-Hyland EF (1996) The influence of spousal social support on psychological and physical health among persons with type II diabetes mellitus: A test of the optimal matching model of social support. Unpublished dissertation, University of Iowa, Iowa City, IA.

[28] John OP, Gross JJ (2007) Individual differences in emotion regulation. In J.J. Gross (Ed.), Handbook of emotion regulation. New York: Guilford Press.

[29] Tijhuis MAR, Flap HD, Foets M, Groenewegen PP (1995) Social support and stressful life events in two dimensions: Life events and illness as an event. Soc Sci Med 40:1513–1526 doi:10.1016/0277-9536(94)00276-Y. 7667656

[30] Jacobsen DE (1986) Types and timing of social support. J Health Soc Behav 27:250–264 doi:10.2307/2136745. 3772062

[31] Weiss RS (1976) Transition states and other stressful situations: Their nature and programs for their management. In: Caplan G, Killilea Meditors.Support Systems Mutual Help: Multi-disciplinary and Explorations. New York: Grune and Stratton. pp.213–232.

[32] Pennebaker JW (1997) Writing about emotional experiences as a therapeutic process. Psychol Sci 8(3):162–166 doi:10.1111/j.1467-9280.1997.tb00403.x.

[33] Pennebaker JW, Beall SK (1986) Confronting a traumatic event: Toward an understanding of inhibition and disease. J Abnorm Psychol 95(3):274–281 doi:10.1037/0021-843X.95.3.274. 3745650

[34] Pennebaker JW, Chung CK (2007) Expressive writing, emotional upheavals, and health. In: Friedman HS, Cohen Silver Reditors.Foundations of Health Psychology.New York: Oxford University Press. pp.263–284.

[35] Pennebaker JW, Kiecolt-Glaser JK, Glaser R (1988) Disclosure of traumas and immune function: health implications for psychotherapy. J Consult Clin Psychol 56:239–245 doi:10.1037//0022-006X.56.2.239. 3372832

[36] Peterkin AD, Prettyman AA (2009) Finding a voice: revisiting the history of therapeutic writing. Med Humanit 35:80–88 doi:10.1136/jmh.2009.001636. 23674701

[37] Smyth JM, Stone AA, Hurewitz A, Kaell A (1999) Effects of writing about stressful experiences on symptom reduction in patients with asthma or rheumatoid arthritis: A randomized trial. JAMA 281(14):1304–1309 doi:10.1001/jama.281.14.1304. 10208146

[38] Lepore SJ, Greenberg MA, Bruno M, Smyth JM (2002) Expressive writing and health: Self-regulation of emotion-related experience, physiology, and behavior. In: Lepore SJ, Smyth J, editors.The writing cure: How expressive writing promotes health and emotional well-being.Washington, DC: American Psychological Association. pp.99–117.

[39] Lu Q, Stanton AL (2010) How benefits of expressive writing vary as a function of writing instructions, ethnicity and ambivalence over emotional expression. Psychol Health 25(6):669–684 doi:10.1080/08870440902883196. 20204944

[40] Goldsmith DJ, McDermott VM, Alexander SC (2000) Helpful, supportive, and sensitive: Measuring the evaluation of enacted support in personal relationships. J Soc Pers Relat 17:369–391 doi:10.1177/0265407500173004.

[41] Clark RA, Pierce AJ, Finn K, Hsu K, Toosley A, et al (1998) The impact of alternative approaches to comforting, closeness of relationship, and gender on multiple measures of effectiveness. Cent States Speech J 49:224–239 doi:10.1080/10510979809368533.

[42] Jones SM, Wirtz J (2006) How does the comforting process work? An empirical test of an appraisal-based model of comforting. Hum Commun Res 32:217–243 doi:10.1111/j.1468-2958.2006.00274.x.

[43] Pennebaker JW (1982) The psychology of physical symptoms. New York: Springer-Verlag.

[44] Zijlstra H, Van Meerveld T, Van Middendorp H, Pennebaker JW, Geenen RD (2004) De Nederlandse versie van de “Linguistic Inquiry and Word Count”(LIWC). Gedrag Gezond 32: 271–281.

[45] Pennebaker JW (1993) Putting stress into words: Health, linguistic, and therapeutic implications. Behav Res Ther 31:539–548 doi:10.1016/0005-7967(93)90105-4. 8347112

[46] Pennebaker JW, Francis ME (1996) Cognitive, emotional, and language processes in disclosure. Cogn Emot 10(6):601–626 doi:10.1080/026999396380079.

[47] Tausczik YR, Pennebaker JW (2009) The psychological meaning of words: LIWC and computerized text analysis methods. J Lang Soc Psychol 29(1):24–54 doi:10.1177/0261927X09351676.

[48] Vlachopoulos SP, Michailidou S (2006) Development and initial validation of a measure of autonomy, competence, and relatedness in exercise: The basic psychological needs in exercise scale. Meas Phys Educ Exerc Sci 10(3):179–201 doi:_ 10.1207/s15327841mpee10034.

[49] Holmes TH, Rahe RH (1967) The social readjustment rating scale. J Psychsom Res 11:213–218. 10.1016/0022-3999(67)90010-4 6059863

[50] Cochrane R, Robertsen A (1973) The Life Events Inventory: a measure of the relative severity of psychological stresors. J Psychsom Res 17:135–139. 10.1016/0022-3999(73)90014-7 4741684

[51] Linden W (1984) Development and initial validation of a life event scale for students. Canadian Counsellor 18(3):106–110.

[52] American Psychological Association Ethics Committee 2010 Amendments to the 2002 “Ethical principles of psychologists and code of conduct”. Am Psych 65(5):493 doi:10.1037/a0020168. 20642307

[53] EU legislation on data protection**.** Available: http://ec.europa.eu/justice/data-protection/. Accessed 2013 Oct 09. (Archived by WebCite at http://www.webcitation.org/6KEjYUQEE)

[54] Dutch legislation on data protection**.** Available: http://wetten.overheid.nl/BWBR0011468/geldigheidsdatum_11-07-2013. Accessed 2013 Oct 09. (Archived by WebCite at http://www.webcitation.org/6KEjjpGbK)

[55] John OP, Gross JJ (2004) Healthy and unhealthy emotion regulation: Personality processes, individual differences, and life span development. J Pers 72:1301–1334 doi:10.1111/j.1467-6494.2004.00298.x. 15509284

[56] Low CA, Stanton AL, Danoff-Burg S (2006) Expressive disclosure and benefit finding among breast cancer patients: Mechanisms for positive health effects. Health Psych 25(2):181–189 doi:10.1037/0278-6133.25.2.181. 16569109

[57] Rimé B, Finkenauer C, Luminet O, Zech E, Philippot P, et al (1998) Social sharing of emotion: New evidence and new questions. European Review of Social Psychology 9:145–189 doi:10.1080/14792779843000072.

[58] Jackson S, Jacobs S (1983) Generalizing about messages: Suggestions for design and analysis of experiments. Hum Commun Res 9(2):169–191 doi:10.1111/j.1468-2958.1983.tb00691.x.

[59] Tamres LK, Janicki D, Helgeson VS (2002) Sex differences in coping behavior: A meta-analytic review and an examination of relative coping. Pers Soc Psychol Rev 6(1):2–30 doi:_ 10.1207/S15327957PSPR06011.

