# Supplementary material for: An Experimental Study on the Effectiveness of Disclosing Stressful Life Events and Support Messages: When Cognitive Reappraisal Support Decreases Emotional Distress, and Emotional Support Is Like Saying Nothing at All
Source: PLoS One. 2014 Dec 22;9(12):e114169. doi: 10.1371/journal.pone.0114169 (PMC4273978; doi:10.1371/journal.pone.0114169)
Supplement: S1 Appendix — Survey questions outcome measures. Symptom/emotion checklist: a state measure (Pennebaker, 1982). (DOCX) [file pone.0114169.s001.docx]

**Appendix S1. Survey questions outcome measures**

Symptom/emotion checklist: a state measure (Pennebaker, 1982)

Right now, at this moment I am experiencing:

|  | Not at all |  |  |  | Very much |
| --- | --- | --- | --- | --- | --- |
| A headache |  |  |  |  |  |
| Watering eyes |  |  |  |  |  |
| Racing heart |  |  |  |  |  |
| Congested nose |  |  |  |  |  |
| Tense muscles |  |  |  |  |  |
| Upset stomach |  |  |  |  |  |
| Flushed face |  |  |  |  |  |
| Sweaty hands |  |  |  |  |  |
| Shortness of breath |  |  |  |  |  |
| Cold hands |  |  |  |  |  |
| Dizziness |  |  |  |  |  |
| Ringing in ears |  |  |  |  |  |

Right now, at this moment, I am feeling:

|  | Not at all |  |  |  | Very much |
| --- | --- | --- | --- | --- | --- |
| Happy |  |  |  |  |  |
| Anxious |  |  |  |  |  |
| Angry |  |  |  |  |  |
| Guilty |  |  |  |  |  |
| Sad |  |  |  |  |  |

Response Evaluations Scale

1. Did you perceive the reaction of the other person to your story as appropriate?
2. Did you perceive the reaction of the other person to your story as pleasant?
3. Did you perceive the reaction of the other person to your story as supportive?

Perceived Relatedness Scale (Vlachopoulos & Michailidou, 2006)

1. I feel that I associate with the person who read and responded to my story, in a very friendly way.
2. I feel extremely comfortable with the other person who read and responded to my story.
3. I feel there are open channels of communication with the other person who read and responded to my story.
4. I feel very much at ease with the other person who read and responded to my story.
