# Supplementary material for: An Experimental Study on the Effectiveness of Disclosing Stressful Life Events and Support Messages: When Cognitive Reappraisal Support Decreases Emotional Distress, and Emotional Support Is Like Saying Nothing at All
Source: PLoS One. 2014 Dec 22;9(12):e114169. doi: 10.1371/journal.pone.0114169 (PMC4273978; doi:10.1371/journal.pone.0114169)
Supplement: S2 Appendix — Codebook Story Subjects. (DOCX) [file pone.0114169.s002.docx]

Appendix S2. Codebook Story Subjects

- - - 1. Death of a family member or close friend
      2. Personal serious problems (divorce (of parents), major legal problems)
      3. Personal illness/injury/being psychologically harmed
      4. Serious issues among significant others or sudden major responsibilities for significant others (illness, legal problems, serious harassment)
      5. Serious argument with family member, girl- or boyfriend, close friend, letting go of a dream
      6. Increased workload, study issues, moving houses, minor legal issues, travelling alone
